# The association between neighborhood economic hardship, the retail food environment, fast food intake, and obesity: findings from the Survey of the Health of Wisconsin

**DOI:** 10.1186/s12889-015-1576-x

**Published:** 2015-03-13

**Authors:** Michael Laxy, Kristen C Malecki, Marjory L Givens, Matthew C Walsh, F Javier Nieto

**Affiliations:** Department of Population Health Sciences, University of Wisconsin Medical School, 610 North Walnut Street, Madison, WI 53726 USA; Institute of Health Economics and Health Care Management, Helmholtz Zentrum München, German Research Center for Environmental Health, Ingolstädter Landstraße 1, 85674 Neuherberg, Germany

**Keywords:** Obesity, Retail food environment, Economic hardship, Fast food consumption

## Abstract

**Background:**

Neighborhood-level characteristics such as economic hardship and the retail food environment are assumed to be correlated and to influence consumers’ dietary behavior and health status, but few studies have investigated these different relationships comprehensively in a single study. This work aims to investigate the association between neighborhood-level economic hardship, the retail food environment, fast food consumption, and obesity prevalence.

**Methods:**

Linking data from the population-based Survey of the Health of Wisconsin (SHOW, n = 1,570, 2008–10) and a commercially available business database, the Wisconsin Retail Food Environment Index (WRFEI) was defined as the mean distance from each participating household to the three closest supermarkets divided by the mean distance to the three closest convenience stores or fast food restaurants. Based on US census data, neighborhood-level economic hardship was defined by the Economic Hardship Index (EHI). Relationships were analyzed using multivariate linear and logistic regression models.

**Results:**

SHOW residents living in neighborhoods with the highest economic hardship faced a less favorable retail food environment (WRFEI = 2.53) than residents from neighborhoods with the lowest economic hardship (WRFEI = 1.77; p-trend < 0.01). We found no consistent or significant associations between the WRFEI and obesity and only a weak borderline-significant association between access to fast food restaurants and self-reported fast food consumption (≥2 times/week, OR = 0.59-0.62, p = 0.05-0.09) in urban residents. Participants reporting higher frequency of fast food consumption (≥2 times vs. <2 times per week) were more likely to be obese (OR = 1.35, p = 0.06).

**Conclusion:**

This study indicates that neighborhood-level economic hardship is associated with an unfavorable retail food environment. However inconsistent or non-significant relationships between the retail food environment, fast food consumption, and obesity were observed. More research is needed to enhance methodological approaches to assess the retail food environment and to understand the complex relationship between neighborhood characteristics, health behaviors, and health outcomes.

**Electronic supplementary material:**

The online version of this article (doi:10.1186/s12889-015-1576-x) contains supplementary material, which is available to authorized users.

## Background

Obesity is a major public health problem in the United States owing to its substantial mortality and morbidity and increased health care costs [1,2]. In response to the increasing rates of obesity in all subgroups of the population, particularly among socially and economically deprived subpopulations, researchers have begun to analyze how people’s social and physical environment might influence eating behavior, activity level, and weight status [3,4]. The retail food and built environments have been identified as key components of the “obesogenic” environment, which might constitute an important determinant of the obesity epidemic [5]. Even though the literature is not entirely consistent, there is growing evidence that physical access to different kinds of food outlets significantly influences dietary patterns and weight status at the population level [6-19]. Previous studies have found that a high density of fast food restaurants in neighborhoods was associated with regular consumption of fast food and a higher prevalence of obesity [6,13-15,18]. In recent studies in Canada and the US, the “Retail Food Environment Index,” defined as the ratio of the number of healthy vs. unhealthy food outlets within a certain boundary around the consumer’s residence, was associated with obesity, thus supporting the hypothesis that the retail food environment has an impact on weight status [11,12]. Previous research further indicated that socially and economically deprived subgroups of the population often face a less favorable retail food environment, which in turn might increase the likelihood for poor quality and energy dense nutrition, and obesity [20-26]. Accordingly, it can be hypothesized that the imbalanced distribution of food outlets might be a contributor to the inequality of obesity among ethnic minorities and different socioeconomic groups.

To date, many studies separately analyzed the associations between neighborhood level deprivation and the retail food environment [20-25] or between the retail food environment and either dietary behavior or weight status [6,11-15,18]. However, none of them simultaneously analyzed these different associations in a single study. In addition, most previous studies have relied on self-reported height and weight and almost none used a population-based sample that represents a large geographic area including both rural and urban populations.

This study aims to fill these gaps by linking data from the statewide, representative Survey of the Health of Wisconsin (SHOW), a commercial business database inventorying food retailers, and socioeconomic data from the US National Census, in order to address the following questions: 1) whether neighborhood-level economic hardship is associated with the quality of the retail food environment; and 2) whether the characteristics of the retail food environment are associated with self-reported frequency of fast food consumption and obesity prevalence in a statewide representative sample.

## Methods

### Data sources and measures

**Figure 1:**
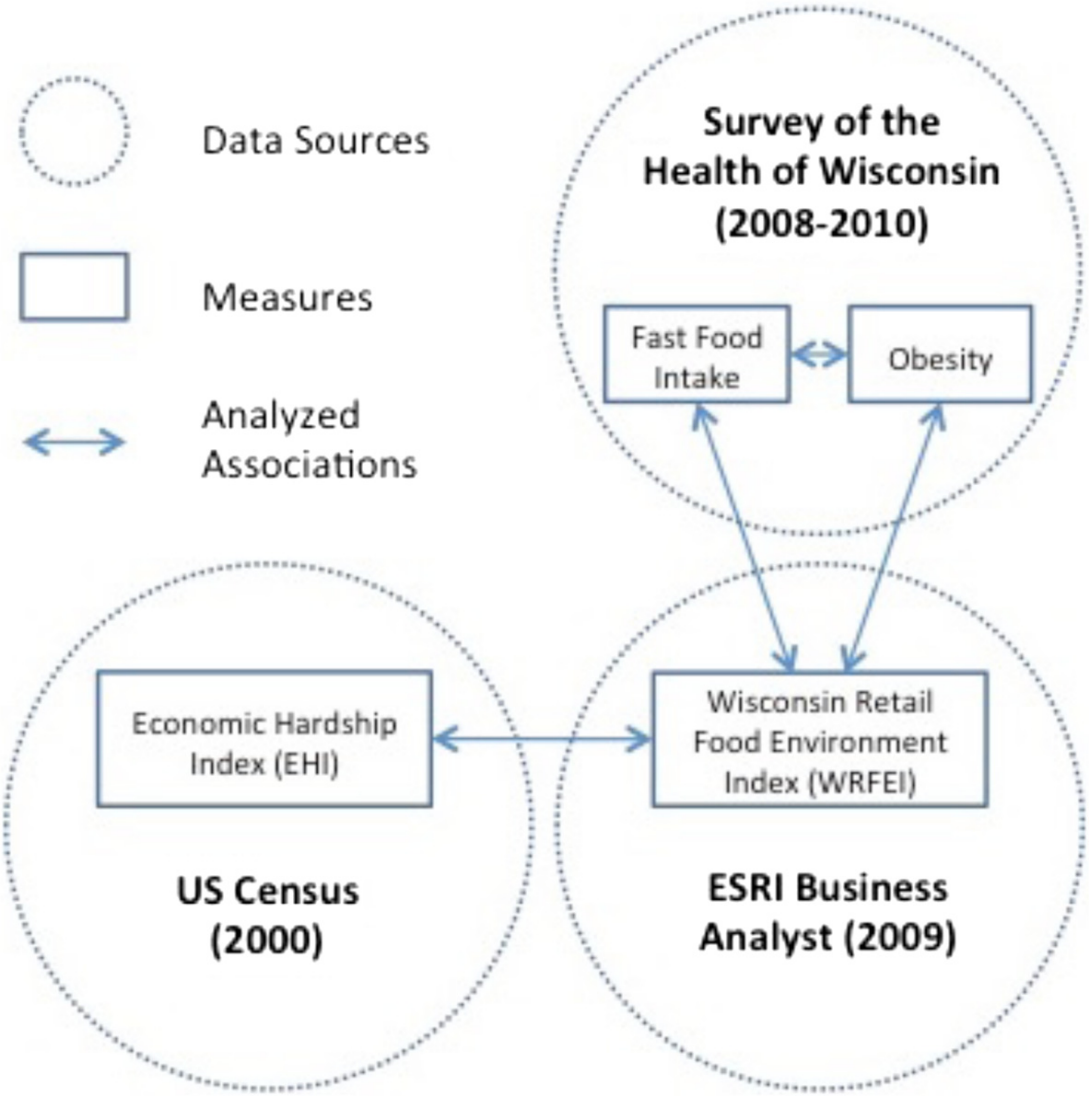
**Study overview describing data sources, measures and analyzed associations.**

A graphical overview of the data sources, measures and analyzed associations for this study is provided in Figure 1. Each of the main data sources is briefly described in the following paragraphs.

#### Health survey data

A probabilistic sample of 1570 Wisconsin adults aged 21–74 years old recruited by the Survey of the Health of Wisconsin (SHOW) between 2008 and 2010 were included in this study. Rationale for the survey, sampling procedure, recruitment methods and the data collection process have been described in detail elsewhere [27]. In brief, the SHOW is an annual cross-sectional survey providing comprehensive data on health and health determinants including physical and mental health history, demographics, behavioral, lifestyle, and household characteristics. The selection of the SHOW participants is based on a two-stage cluster sampling approach. Data were collected in three steps. Demographic, housing and socioeconomic characteristics and physical activity data were collected in an initial face-to-face interview. Dietary habits were gathered using a self-administered questionnaire and weight and height status were measured as part of a physical exam. The study was approved by the UW-Madison Health Sciences Institutional Review Board.

#### Neighborhood-level economic hardship

The Economic Hardship Index (EHI) developed by the Rockefeller Institute of Government was derived from 2000 census data. The EHI measures the social and economic conditions of communities on a block group level using six indicators including:

- crowded housing (percent of housing units with more than 1 person per room),
- poverty (percent of households living below the federal poverty level),
- unemployment (percent of persons over the age of 16 years that are unemployed),
- education (percent of persons over the age of 25 years without a high school education),
- dependency (percent of population that is under age 18 or over age 64 years),
- income level (median per capita).

High indices represent higher neighborhood-level economic hardship. Individuals were classified into quartiles from lowest economic hardship (1st quartile, lowest neighborhood-level deprivation) to highest economic hardship (4th quartile, highest neighborhood-level deprivation) based on census block group level estimates of EHI [26,28].

#### Food retailer database and classification

The ESRI business analyst dataset (2009) was used to define the retail food environment. Food outlets were identified using North American Industry Classification System (NAICS) codes including those starting with 722*** (Food Services and Drinking Places), 4451** (Grocery and Convenience Stores), 4452** (fruit and vegetable markets) and 452111 (department stores). Using previously established criteria, the following three different types of restaurants and food retailers were defined: a) fast food and fast casual restaurants (henceforth designated ‘fast food restaurants’); b) convenience stores including small grocery stores and corner stores (‘convenience stores’); and c) supermarkets, grocery stores, supercenters, produce vendors and farmer markets (‘supermarkets’) [19]. The process of food retailer classification is described in detail in Additional file 1.

#### Accessibility to fast food restaurants, convenience stores and supermarkets

All SHOW households and categorized food outlets were geocoded with ArcGIS North America Geocode Service 10.0. In a second step, the mean distances on a street network from each SHOW selected household to the three closest: a) ‘fast food restaurants;’ b) ‘convenience stores;’ and c) ‘supermarkets,’ were calculated as proxies for accessibility using ArcGIS Network Analyst (ESRI, Redlands, CA).

#### Wisconsin Retail Food Environment Index

To obtain an overall proxy for the retail food environment, an index measure, the Wisconsin Retail Food Environment Index (WRFEI), was calculated. The WRFEI was defined as the ratio of the mean distance to the three closest stores that are assumed to potentially provide “healthier” food options (‘supermarkets’) to the mean distance of stores that are generally assumed to provide fewer healthy food options (‘convenience stores’ and ‘fast food restaurants’). The WRFEI was developed as a variant to previous retail environment indices that were based on counts of retail venues within a certain arbitrarily defined area (zip code, county, etc.) or buffer zone [11,12,19] and it is thought to more accurately reflect the relative quality of the food environment in proximity to the individuals’ homes. High WRFEI values indicate a potentially “unhealthy” retail food environment.

#### Fast food consumption

The total weekly frequency of fast food consumption was derived from a questionnaire asking for the number of times per week individuals eat at or take food from a fast food restaurant/fast casual restaurant for breakfast, lunch, or dinner. As in previous work, intake of fast food ≥2 times a week was defined as ‘regular fast food consumption’ [29].

#### Obesity

The Body Mass Index (BMI) was calculated from measured weight (kg) divided by height (m)^2^. Participants with a BMI ≥30 were classified as obese [30].

#### Covariates

Urbanicity levels were classified based on the Rural–urban Commuting Area Codes (RUCA) available from the Rural Health Research Center (urban: RUCA codes starting with 1, suburban: RUCA codes starting with 2, rural: RUCA codes starting with 3 or higher) [31]. Other covariates were categorized using *a priori* defined criteria, included age (21–39, 40–55, >55 years), annual income (<$25k, $25k-$50k, >$50k), and education (no high school, high school, some college, college degree). Levels of physical activity were defined based on metabolic equivalent (MET) minutes per week, which were derived from self-reported information about light-intensity, moderate-intensity and vigorous-intensity physical activity (<600, 600–2999, ≥3000 MET-min/week) [32].

### Statistical analysis

Analyses were conducted using SAS version 9.2 (SAS Institute Inc, Cary, North Carolina). Descriptive statistics and regression models were adjusted for SHOW cluster sampling design using sampling weights (PROC SURVEYFREQ, PROC SURVEYREG, and PROC SURVEYLOGISTIC). DOMAIN statements were used to stratify the models by urbanicity—with the suburban category excluded from some analyses because of small sample size.

The relationship between neighborhood-level economic hardship and the retail food environment was analyzed using linear regression models, with EHI categorized into quartiles. Contrast-comparisons of WRFEI least square means (LSMEANS) were performed to analyze differences between the 1st (least deprived), the 2nd, the 3rd and the 4th (most deprived) EHI-quartile.

Logistic regression was used to estimate the odds ratio of individual’s obesity status and regular fast food consumption as a function of the food environment predictors. As the distribution of the food environment predictors were skewed, for these analyses, the variables were defined as 3-level ordinal variables: Access to fast food restaurants, supermarkets or convenience stores was categorized into ‘high access’ (tertile of individuals with lowest distance to these kind of food retailers), ‘medium access’ (those in the middle tertile), and ‘low access’ (tertile of individuals with highest distance). Accordingly, the retail food environment was classified into ‘unfavorable’ (tertile of highest WRFEI values), ‘medium’ (tertile of middle WRFEI values) and ‘favorable’ (tertile of lowest WRFEI values). All models were controlled for gender, age, race/ethnicity, education, and income and were reported stratified according to urban/rural status. Models predicting odds of obesity were additionally adjusted for level of physical activity. Using the same set of covariates we further tested the association between fast food consumption and obesity. For this, we applied a logistic regression model with regular fast food consumption vs. no regular fast consumption as a dichotomous predictor variable.

#### Sensitivity analyses

In the absence of a definitely established gold standard for the assessment of the retail food environment, we performed several sensitivity analyses using different definitions to verify the robustness of our results. Thus, we calculated the retail food environment index (RFEI) as proposed by Spence et al., which is defined by the equation ‘RFEI = (F + C)/G’, where ‘F’, ‘C’, and ‘G’ represent the number of fast food restaurants, convenience stores, and grocery stores, respectively, within a buffer zone of 1600 m (approximately 1 mile), independent of the real distance on the street network [11]. Furthermore, in order to assess if the examined associations in our study are sensitive to the number of closest food retailers captured by the retail food environment proxies, we used an alternative definition of the retail food environment that was based on the distances to the (one) closest food retailer and also to the five closest food retailers. Finally, we also tested if results were sensitive to the threshold used to define ‘regular fast food consumption’.

## Results

### Characteristics of the study population

Characteristics of the SHOW sample are presented in Table 1. The majority of study participants were white and more than half of them lived in urban settings. The overall obesity prevalence among participants was 38%, and 46% of the sample reported to eat fast food at least twice a week. Obesity prevalence was similar in both genders, but higher for people of older ages, those less educated, of racial/ethnic minorities, and inactive people. The frequency of fast food consumption was higher for people of younger ages and among African Americans and Hispanics (results not shown).

**Table 1:** **Descriptive characteristics of the study sample**

		**N**	**% population**
		1570	100.0
Gender	men	707	50.2
	women	863	49.8
Age	21-39	516	37.9
	40-55	538	34.5
	>55	516	27.6
Race/ethnicity	White	1362	84.9
	African American	90	6.2
	Hispanic	54	3.8
	Other	59	5.1
Income	low (<$25k)	536	34.7
	medium ($25-50k)	553	35.6
	high (>$50k)	442	29.7
Education	no high school	126	8.9
	high school	321	20.9
	some college	603	37.2
	college degree	517	33.1
Urbanicity	urban	757	50.4
	suburban	193	11.8
	rural	620	37.8
Physical activity	<600 MET-min/week	594	39.4
	600-2999 MET-min/week	342	37.7
	≥3000 MET-min/week	634	22.9
Weight status	obese (BMI ≥ 30)	525	37.8
Fast food consumption	≥2 times a week	585	46.4

### Characteristics of the retail food environment

**Figure 2:**
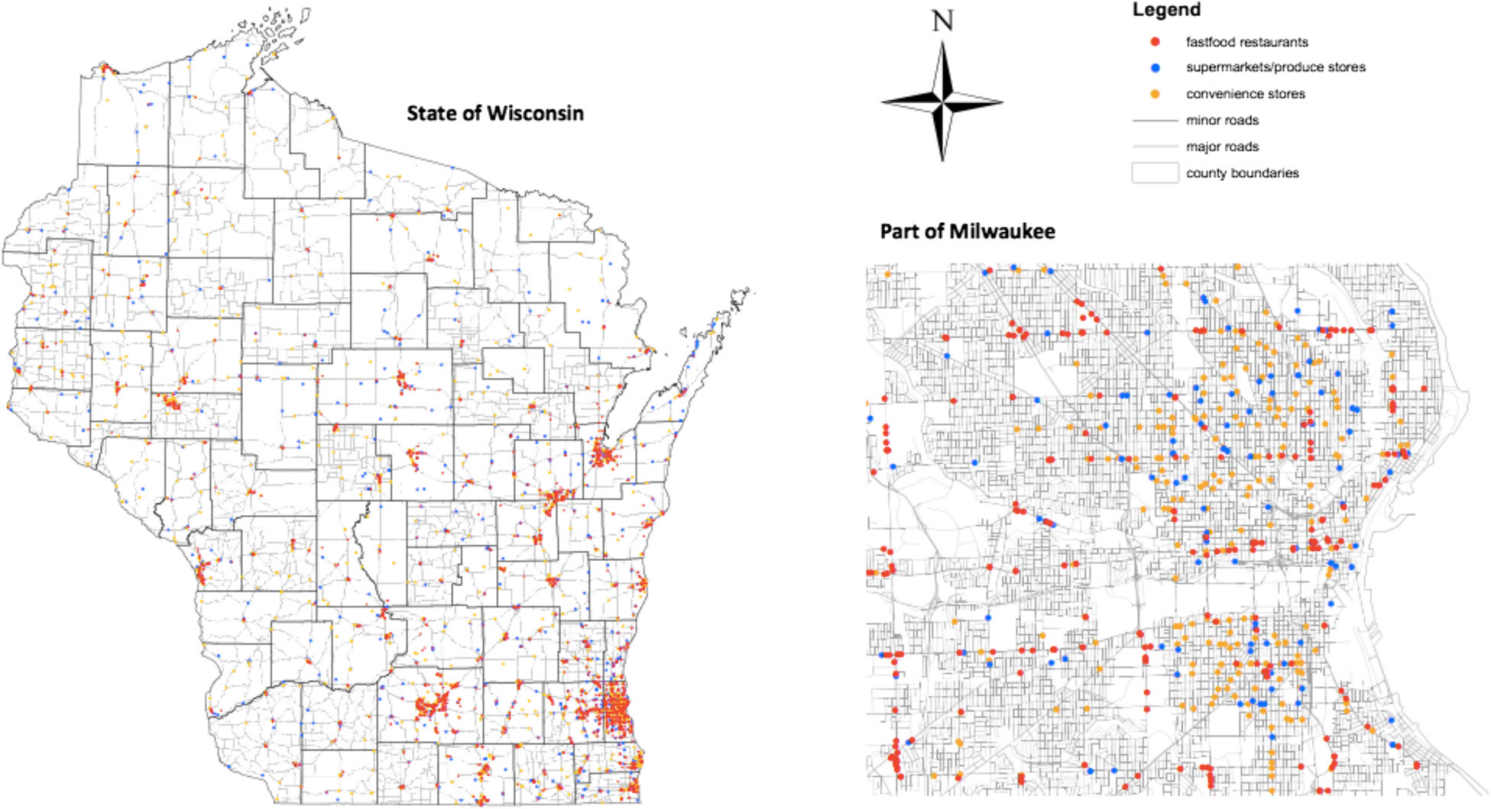
**Geographical distribution of food outlets in Wisconsin.** Note: The inset represents the Milwaukee metropolitan area.

The mean-distance of the SHOW participants to the three closest fast food restaurants, convenience stores, and supermarkets was, on average, 4.5 km, 5.5 km and 5.4 km, respectively. A similar pattern was observed after stratifying into urban, suburban and rural areas, although, as expected, the mean distances to food outlets in urban areas were generally smaller than in suburban and rural areas. The overall mean WRFEI was 2.32 (urban 2.16; rural 2.80) indicating that, on average, the mean-distance to the three closest potentially healthy food retailers (‘supermarkets’) was more than twice that of the mean distance to the three closest potentially unhealthy food retailers (‘fast food restaurants’ or ‘convenience stores’). The overall distribution of food outlets is illustrated in Figure 2.

### Association between economic hardship and the WRFEI

Table 2 describes the relationship between the economic hardship index and the retail food environment. In general, WRFEI increased with increasing level of economic hardship. The p-value for the linear trend test was statistically significant in the overall model, as well as in the urban and rural strata (all p < 0.01), although the relation was not strictly linear, particularly in the rural stratum. The results were similar when the log-transformed WRFEI (geometric mean) was used (results not shown).

**Table 2:** Linear regression analysis model:* Means of the Wisconsin Retail Food Environment Index (WRFEI) according to the level of neighborhood-level economic hardship, overall and stratified for urbanicity

**Economic Hardship Index (EHI)**	**Wisconsin Retail Food Environment Index (WFREI)**								
	**Overall**			**Urban**			**Rural**		
	**(n = 1570)**			**(n = 757)**			**(n = 620)**		
	**Mean**	**p-value**		**Mean**	**p-value**		**Mean**	**p-value**	
1st quartile: (least deprived)	1.77	ref.		1.94	ref.		1.22	ref.	
2nd quartile:	2.14	vs. 1st	0.05	1.66	vs. 1st	0.10	2.09	vs. 1st	0.06
3rd quartile:	2.89	vs. 1st	<0.001	2.19	vs. 1st	0.18	3.21	vs. 1st	<0.001
4th quartile: (most deprived)	2.53	vs. 1st	<0.001	2.91	vs. 1st	<0.001	2.14	vs. 1st	0.04
Linear trend test			<0.001			<0.001			0.001

*Linear regression with least square means estimating the association between the Economic Hardship Index (EHI) and the Wisconsin Retail Food Environment Index (WRFEI).

### Association between access to food outlets and obesity

In Table 3, each cell represents a separate logistic regression model on the relation between accessibility to each type of food outlet (defined based on mean distance from participants’ residence), as well as tertiles of WRFEI and odds of obesity. Residents in urban areas with medium and low access to convenience stores are three times (p < 0.01) and two times (p < 0.05) respectively more likely to be obese than individuals with high access. However, this relationship was not apparent among residents in rural areas or when both rural and urban were combined. In general, confidence intervals around odds ratios were large and no consistent or significant trends were observed in the relationship between access to supermarkets or the WRFEI tertiles and the odds of obesity.

**Table 3:** **Logistic regression analysis: Adjusted Odds Ratios (AOR) of obesity according to access to food outlets, overall and stratified for urbanicity**

		**Overall** ^**a)**^		**Urban** ^**b)**^		**Rural** ^**b)**^	
		**(n = 1570)**		**(n = 757)**		**(n = 620)**	
**Accessibility**		**AOR**	**95% CI**	**AOR**	**95% CI**	**AOR**	**95% CI**
Fast food restaurants	high	1		1		1	
	medium	1.04	(0.73, 1.49)	1.09	(0.61, 1.94)	0.66	(0.38, 1.13)
	low	0.90	(0.55, 1.48)	0.87	(0.54, 1.41)	0.87	(0.87, 1.40)
Convenience stores	high	1		1		1	
	medium	1.23	(0.83, 1.85)	3.24**	(1.73, 6.08)	0.76	(0.42, 1.41)
	low	0.99	(0.60, 1.63)	2.11*	(1.15, 3.89)	1.40	(0.85, 2.33)
Supermarkets	high	1		1		1	
	medium	0.94	(0.66, 1.34)	0.97	(0.57, 1.63)	0.62	(0.35, 1.13)
	low	1.06	(0.65, 1.72)	1.29	(0.79, 2.11)	0.98	(0.56, 1.72)
WRFEI	favorable	1		1		1	
	medium	1.04	(0.70, 1.54)	1.28	(0.78, 2.11)	0.68	(0.38, 1.20)
	unfavorable	1.13	(0.75, 1.70)	1.46	(0.81, 2.65)	0.73	(0.42, 1.30)

OR odds ratio, CI confidence interval, WRFEI Wisconsin Retail Food Environment Index;

high access: tertile of participants with smallest mean-distance to 3 closest retailers;

medium access: tertile of participants with medium mean-distance to 3 closest retailers;

low access: tertile of participants with greatest mean-distance to 3 closest retailers;

^a)^each model is adjusted for gender, age, race/ethnicity, education, income, physical activity and urbanicity;

^b)^each model is adjusted for gender, age, race/ethnicity, education, income, and physical activity.

*p < 0.05 **p < 0.01.

### Association between access to fast food outlets and fast food consumption

The odds ratios for regular fast food consumption according to access to the fast food restaurants are shown in Table 4. In general, lower access to fast food restaurants was weakly associated with a lower prevalence of regular fast food consumption. Compared to urban residents with a high access to fast food restaurants, those with medium or with low access to fast food restaurants had a 38% (p < 0.05) and 41% (p = 0.09) reduced odds of consuming fast food at least twice per week, respectively. Although additional analyses showed that this association was not sensitive to the choice of the cut-off definition for ‘regular fast food consumption’ (i.e., ≥3 times/week) it needs to be emphasized that uncertainty around effect estimates was rather large in all models.

**Table 4:** **Logistic regression analysis: Adjusted Odds Ratios (AOR) of individuals’ reported regular fast food consumption (≥2 times/week) according to access to fast food restaurants, overall and stratified for urbanicity**

		**Overall** ^**a)**^		**Urban** ^**b)**^		**Rural** ^**b)**^	
		**(n = 1570)**		**(n = 757)**		**n = (620)**	
**Accessibility**		**AOR**	**95% CI**	**AOR**	**95% CI**	**AOR**	**95% CI**
Fast food restaurants	high	1		1		1	
	medium	0.83	(0.58, 1.18)	0.62*	(0.38, 1.00)	0.78	(0.42, 1.44)
	low	0.78	(0.52, 1.16)	0.59	(0.32, 1.08)	0.80	(0.43, 1.49)

OR odds ratio, CI confidence interval, WRFEI Wisconsin Retail Food Environment Index;

high access: tertile of participants with smallest mean-distance to 3 closest retailers;

medium access: tertile of participants with medium mean-distance to 3 closest retailers;

low access: tertile of participants with greatest mean-distance to 3 closest retailers;

^a)^each model is adjusted for gender, age, race/ethnicity, education, income and urbanicity;

^b)^each model is adjusted for gender, age, race/ethnicity, education and income;

*p < 0.05.

### Association between fast food consumption and obesity

Table 5 shows the odds for obesity according to the reported frequency of fast food consumption. When the latter was used as a continuous variable, we found a statistically significant association, with an 8% increase in the probability of obesity per each meal of fast food per week (p < 0.01). Participants reporting 2 or more fast food meals per week had a 35% higher odds of obesity than those consuming fast food meals less than twice per week, a difference that was marginally statistically significant (p = 0.06).

**Table 5:** **Logistic regression analysis: Adjusted Odds Ratios (AOR) of obesity according to alternative definitions of fast food consumption**

	**Continous model** ^**a)**^			**Categorial model** ^**a)**^		
	**Predictor**	**AOR**	**95% CI**	**Predictor**	**AOR**	**95% CI**
Fast food consumption	# of meals per week	1.08**	(1.02, 1.14)	<2 meals per week	1	
				≥2meals per week	1.35	(0.99, 1.84)

OR Odds Ratio, CI Confidence interval;

^a)^model is adjusted for gender, age, race/ethnicity, education, income, physical activity and urbanicity;

**p < 0.01.

### Sensitivity analyses

Using the methodological approach of Spence et al. to define the retail food environment [11] yielded a similar association between neighborhood-level economic hardship and the retail food environment; e.g., higher RFEI values in the more deprived EHI-quartiles (results not shown). Categorizing this RFEI into a 3-level ordinal variable and applying this variable in the respective logistic regression model showed that this alternative operationalization of the retail food environment was also not predictive for obesity. Further, applying predictor variables in our study which are based on the mean distance to the one and five closest food retailers (instead to the 3 closest) showed qualitatively comparable patterns of associations in terms of direction and magnitude, compared to using the mean distance to the three closest food retailers. Changing the threshold of regular fast food consumption to at least three times a week was associated with a more than 60% increased odds of being obese (p < 0.01). Finally, when alternative categorization of covariates was used in multivariate models the results were virtually identical to those reported here (not shown).

## Discussion

This study shows that neighborhood economic hardship is associated with an unfavorable retail food environment. We also found a weak indication that higher access to fast food restaurants is associated with a higher likelihood of regular fast food consumption and that fast food consumption is associated with obesity. However, uncertainty around these effect estimates was large and we did not find significant or consistent associations between characteristics of the retail food environment and obesity prevalence.

Previous cross-sectional studies suggest that residents living in low SES areas are exposed to a more unfavorable retail food environment [20-23] and that the availability of food choices is correlated with the residents’ dietary behavior and weight status [6-8,11-15]. The results of our study only partly support the evidence from these studies. We did not find a significant or consistent relationship between the retail food environment or access to fast food restaurants and obesity prevalence. Contrary to our hypothesis, we found an inverse association between the access to convenience stores and obesity and we only observed a borderline significant association between access to fast food outlets and fast food consumption in the urban strata. The reasons for these somehow counterintuitive results or weak associations remain unclear. It might be possible that despite a vast body of research supporting the ‘obesogenic’ environment hypothesis [5,11-15] this might not apply in the analyzed sample of Wisconsin residents. As an alternative explanation, it is possible that these non-significant findings are related to the methodology for the assessment of the retail food environment. In Morland et al.’s study, for example, inconsistent results were found when using density of fast food restaurants/convenience stores (positively associated with obesity) compared to results using distance-based measures similar to those used in our study (negatively associated with obesity) [7]. Measurement error (e.g., business retailer data not being entirely up to date) could result in non-differential misclassification and bias towards the null. In contrast, our results do support previous research indicating that fast-food consumption is associated with obesity [6] and that individuals from socially and economically deprived neighborhoods face a substantially worse retail food environment than residents from less deprived areas [20-22]. This study therefore suggests that, in Wisconsin, the retail food environment might play a smaller role in the etiology of obesity then one could conclude from reviewing the current literature on this topic and that interventions aimed at improving fruit and vegetable consumption among disadvantaged populations should take the retail food environment into account as a potential barrier to successful implementation of behavioral interventions.

To detect potential relationships between the retail food environment, other neighborhood-level characteristics and health outcomes, an accurate assessment of the individual's perceived and real retail food environment is needed. In the current study, we used proxies that are based on the mean distance to the three closest types of food retailers. The choice of this methodological approach is based on the following considerations: In the past, the vast majority of studies used density-based measurements with researcher-defined zones such as zipcode [16], census block [23], census tract [7,18,21], county [14], or state borders [15] or defined boundaries around individuals’ homes, schools or working places [8,13,33,34]. As previously discussed, these approaches may result in bias as consumer behavior might not be limited by artificially assigned boundaries [35]. In addition, density-based measures are predominately designed for use in urban settings and are hardly applicable in heterogeneous settings that include also rural neighborhoods. As observed in this study, rural residents typically do not have a single food retailer within commonly used buffering zones (0.5-1 mile) and therefore cannot meaningfully be assigned to a level of exposure using metrics relevant to urban environments, where the count of different food retailers within this buffering zone might be a meaningful indicator for the retail food environment. Previous studies that used proximity measures from consumer’s homes or schools were mainly based on the distance to the (one) closest food retailer [7,24]. However, as people choose their favorite food retailers based on a variety of reasons including prices, product lines and their personal preferences and resources, proximity to the one closest food retailer may not be the strongest determinant of consumer behavior. Besides this qualitative argument, we hypothesized that a rather smoothed proxy, e.g., averaging the distances to the three closest food outlets, is technically less prone to errors concerning missing retailers in the business food retailer database and concerning the artificial classification of food retail categories.

The strength of this study is the linkage of data from three data sources, including the population-based Survey of the Health of Wisconsin, a business food retailer database, and the US Census. This approach permitted us to comprehensively test cross-sectional associations that are assumed to lie on the causal chain between neighborhood characteristics and objectively measured weight status. In addition, the development and application of a new methodological approach to define the retail food environment, which is assumed to be applicable in heterogeneous geographic settings made it possible to test these associations in a large representative sample of Wisconsin residents.

The study has some limitations that are worth mentioning. Although the various sensitivity analyses indicated that the methodology used is quite robust and the application of an alternative index measure for the retail food environment led to comparable results, the accuracy and validity of the proxies to define the retail food environment might be limited. Previous research has also documented the existence of errors in business databases and, despite high geocode matching rates in this study, the occurrence of inaccuracies in the geocoding process cannot be excluded [36,37]. Another possible limitation stems from the use of a business food retailer database following the approach used in previous studies [11,12,19]. Because the retail food environment is constantly changing, it is virtually impossible to obtain an entirely accurate and up-to-date measure. Further, the categorization of food outlets might also result in some degree of misclassification, especially since no direct inspection of the food outlets was carried out. Finally, we cannot rule out the occurrence of residual confounding, e.g., food retailer density in urban areas might possibly be correlated with walkability. However, the majority of the limitations listed above are expected to induce non-differential errors that would typically tend to bias the results towards the null. As such, they could potentially explain some of the negative or weak results, but they are unlikely to explain the significant associations. In the interpretation of the results, the effect of reverse causation needs to be considered; namely, that the relationship between the food environment and eating behavior is co-determined by the food industry’s business strategies in response to consumer behavior and individuals’ residential preferences. Finally, the social context and social norms driving individuals’ behaviors and food preferences were not considered in this analysis.

## Conclusion

This study explores the question of how the social and retail food environment influence fast-food consumption and obesity within a diverse study population of Wisconsin residents. We found that high access to fast food outlets was weakly associated with regular fast food consumption and regular fast food consumption is in turn weakly associated with obesity. However, the former association is only apparent in the urban subsample and we also did not find a direct link between the fast food environment and obesity. The strong association between economic hardship and the retail food environment, which was also found in previous studies, implicates that neighborhood-level deprivation should be considered as a potential barrier for successful implementation of behavioral diet-related interventions. Future research should aim to improve the methodology of assessing the retail food environment to improve the understanding for the interrelation of the physical build environment, neighborhood characteristics and health outcomes.

## Additional file

Additional file 1:
**Classification of food retailers.**
