# Supplementary material for: The association between neighborhood economic hardship, the retail food environment, fast food intake, and obesity: findings from the Survey of the Health of Wisconsin
Source: BMC Public Health. 2015 Mar 13;15:237. doi: 10.1186/s12889-015-1576-x (PMC4409709; doi:10.1186/s12889-015-1576-x)
Supplement: Additional file 1: — Classification of food retailers. [file 12889_2015_1576_MOESM1_ESM.pdf]

Supplementary appendix 1

Classification of food retailers

The categorization of fast food and fast casual restaurants was based on the explanation given to the SHOW participants during the interview on dietary habits, wherein fast food restaurants were described "as chains such as McDonald's and Burger King" and fast casual restaurants "as places that somewhat promise higher quality of food and atmosphere, such as for example Noodles and Company and Panera Bread." Full service restaurants with NAICS codes 72211014, 72211016, 72211019, 72211021 and limited Service Restaurants with the NAICS codes 72221103, 72221104, 72221105, 72221106 met the general criteria for the fast food and fast casual category (n=9492). Restaurants with 5 or more locations were included. Through systematic quality checks of 5 independent raters, this list was further refined to eliminate potential high quality sit-down restaurants. All major fast food chains, such as McDonalds, Burger King or Taco Bell were represented in this final list (n=2680).

Convenience stores were classified under the NAICS code 44512001. All 923 convenience stores listed under this code number were included in this study. Most businesses of this category were small single location convenience stores. The rest of the stores belonged to big chains such as Kwik-Trip, Holiday Station stores and Pick'n Save. Grocery Stores were defined with the NAICS codes 44511001, 44511002, 44511003 and 44511005 (n=1342). In our analysis small single grocery stores (corner stores) were also categorized as convenience stores as it was expected that they also provided a limited variety of food options. Therefore, all "grocery stores" which did not have the word "supermarket" in their business name and which additionally had less than 3 employees and less than $1 million of sale were classified as convenience stores (n=450). Data quality checks via internet research were performed on businesses with this small size, but belonging to a chain or having more than one location to filter misclassification errors of the data source. In all, there were 1390 convenience stores in this study.

All grocery stores and supermarkets with the NAICS code 44511001, 44511002, 44511003 and 44511005 (n=1342), that were not classified as convenience stores according to the criteria described above, were categorized as supermarkets/grocery stores (n=875). In addition, all department stores (NAICS code 45211101), exclusively Wal-Mart Supercenters and Target Supercenters, were added to this group (n=60). A total of 935 grocery stores and supermarkets were included. Produce stores were defined by the code numbers 44523001 and 44523003 (n=129). All 129 listed produce stores were included in the analysis. Farmers' markets were not categorized and listed in the NAICS. Therefore, a dataset of the United States Department for Agriculture including all registered Wisconsin farmers' markets was downloaded (http://www.ams.usda.gov). The source contained the name of the markets, the city, ZIP code and x- and y- coordinates of the actual physical location. 213 farmer markets were included in the analysis.
